# Genome-Wide Association Analysis of Flavor Precursor Traits in Chengkou Mountain Chicken

**DOI:** 10.3390/ani15121726

**Published:** 2025-06-11

**Authors:** Haiwei Wang, Yu Huang, Lingbin Liu, Xin Zhang, Donghang Deng, Zhen Wang, Guangliang Gao, Qigui Wang

**Affiliations:** 1 Institute of Poultry Science, Chongqing Academy of Animal Sciences, Rongchang, Chongqing 402460, China; wanghw@cqaa.cn (H.W.); yibo08051004@163.com (X.Z.); dengdh94@163.com (D.D.); zhwang2022@163.com (Z.W.); 2 College of Animal Science and Technology, Southwest University, Beibei, Chongqing 400715, China; hy0157@163.com (Y.H.); liulb515@163.com (L.L.)

**Keywords:** Chengkou mountain chicken, flavor precursor, GWAS

## Abstract

Chengkou mountain chicken, a Chinese indigenous breed, exhibits unique flavor characteristics. However, the genetic basis of its flavor precursor substances remains unexplored. We performed a genome-wide association study (GWAS) using low-coverage whole-genome sequencing (lcWGS) and genotype imputation to explore genetic markers linked to flavor precursors (nucleotides, amino acids, etc.) in Chengkou mountain chicken breast muscle. We identified 44 SNPs potentially or significantly associated with flavor precursor traits and localized 18 genes. Functional analysis revealed eight important candidate genes, including *ZBTB20*, *RFX4*, *MAMLD1*, *SYN3*, *ABTB3*, *PRPF39*, *LRFN5*, and *DGCR14*, which may play key roles in influencing flavor precursor substances. Moreover, two SNPs residing in the haplotype block (53,448,483 bp to 53,450,834 bp) on chromosome 1 were significantly associated with a di-unsaturated acyl chain (C20:2) residue and mapped to the *ABTB3* gene. This study analyzes the composition of flavor precursor substances in the pectoralis major muscle of Chengkou mountain chicken. Moreover, the SNPs, haplotypes, and candidate genes identified in this study can be used to improve the accuracy of the marker-assisted selection of traits related to flavor precursor substances in the breast muscle of chicken. In addition, the candidate genes that are significantly associated with these traits will potentially lay the foundation for future genetic selection aimed at improving the flavor traits of chicken meat.

## Simple Summary

Chengkou mountain chicken is a local chicken breed in China. However, there is relatively limited research on the genetic basis underlying its flavor precursor compounds. In this study, we determined and analyzed the composition of flavor precursor substances in the pectoral muscle of Chengkou mountain chicken and conducted a genome-wide association analysis to explore the genetic markers related to these flavor precursors. Ultimately, we identified 44 single nucleotide polymorphisms (SNPs) potentially or significantly related to flavor precursor traits and discovered eight key candidate genes that potentially affect flavor precursor substances. These SNPs and candidate genes may lay the foundation for genetic selection to improve the flavor characteristics of chickens in the future.

## 1. Introduction

Meat flavor refers to a complex mixture perceived through smell and taste, including five basic tastes (sweet, savory, bitter, sour, and salty) and aromatic components [1,2]. The formation of characteristic meat flavors fundamentally depends on flavor precursors, which can be categorized into water-soluble and fat-soluble components based on their chemical properties [3]. Water-soluble precursors include amino acids, peptides, sugars, nucleotides, and thiamine, whereas fat-soluble precursors mainly consist of fatty acids and phospholipids [3]. These precursors undergo various processes during cooking—such as the Maillard reaction between amino acids or peptides and reducing sugars, lipid oxidation, and interactions between lipid oxidation products and the Maillard reaction—to generate flavor compounds [2,3,4,5,6,7]. Water-soluble flavor precursors contribute to flavor through Maillard reactions, protein degradation, and caramelization, whereas fat-soluble precursors generate volatile compounds like aldehydes, ketones, and alcohols through lipid oxidation and thermal decomposition, which enhance the aroma of meat [6,8,9].

Genome-wide association studies (GWASs) are a powerful tool for identifying SNPs (single nucleotide polymorphisms) and the functional genes associated with complex traits. GWASs have been crucial in pinpointing genes linked to meat quality and flavor, providing a basis for the genetic improvement in livestock and poultry [10,11,12]. For example, a genomic region containing an SNP (rs316338889) was found on chromosome 5 of Korean native chicken associated with IMP (Inosinic Acid), inosine, and hypoxanthine levels, highlighting candidate genes such as *C5NT1AL*, *DUSP8*, *INS*, *IGF2*, *TNNT2*, and *TNNT3* [10]. Kim et al. conducted a GWAS on the free amino acid and nucleotide traits of F2 chicken populations crossbred from White Leghorn and Yeonsan Ogye, identifying nine important SNPs for amino acids such as arginine, glycine, lysine, and threonine, as well as essential free amino acids. In addition, the researchers identified six important genomic regions with candidate genes including *HOXA3*, *KCNRG*, *KCNIP4*, *MMUT*, and *THSD7B* [11]. Cho et al. also conducted a GWAS on fatty acid traits in the F_2_ chicken population, finding significant SNPs for 15 fatty acid traits primarily located on chromosome 10, associated with genes such as *ACSS3*, *ACSL4*, *BTG1*, *CYB5R4*, *ELOVL4*, *MCEE*, *ME1*, *PPARGC1A*, and *TRPM1* [12].

Compared to high-depth whole-genome sequencing, low-coverage whole-genome sequencing (lcWGS) combined with genotype imputation is cost-effective while still ensuring accuracy in related studies [13,14]. For example, Zhang et al. genotyped 297 Duroc pigs using lcWGS, obtaining 19,306,498 SNPs with an accuracy of 0.984 [15]. Yang et al. conducted a GWAS on 2869 Duroc boars, obtaining 11.3 million SNPs [16]. Huang et al. performed low-depth whole-genome resequencing on Xingguo gray geese and identified 12,490,912 SNPs. They then performed a GWAS for beak length traits, identifying 57 SNPs associated with the trait. These SNPs are found in the genes *ANAPC4*, *CCDC149*, *DHX15*, *LGI2*, *SEPSECS*, *Slc34a2*, and *TAPT1* [17]. Ouyang et al. also used lcWGS to perform a GWAS for wing length traits in 772 Xingguo gray geese at 420 days old, identifying 119 SNPs with significant genome-wide associations located in five regions on chromosome 4. They found that the candidate genes *APBB2*, *GRXCR1*, *NSUN7*, *RBM47*, *SLAIN2*, and *SLC10A4* may play important roles in feather, muscle, and bone growth and development [18].

Meat flavor serves as a critical indicator of meat quality and exerts a significant influence on consumer purchasing behavior and dietary preferences. Local Chinese chicken breeds are well known for their tasty meat and unique flavors, which are highly appreciated by domestic consumers. However, research on the flavor precursors of Chengkou mountain chicken—a local breed in China—remains limited [19]. This study aims to explore the flavor precursor substances (nucleotides, amino acids, fatty acids, and intramuscular fat) in the breast muscle of Chengkou mountain chicken and perform a GWAS based on lcWGS data and genotype imputation to identify SNPs and candidate genes related to flavor precursors in this breed. In addition, linkage disequilibrium analysis and haplotype block validation were performed on the significantly associated SNPs. This study reveals the composition of flavor precursors in Chengkou mountain chicken and provides valuable data for future genetic research and breeding of this breed.

## 2. Materials and Methods

### 2.1. Ethical Statement

This study was approved by the Animal Care and Welfare Committee of the Chongqing Academy of Animal Sciences (license number: XKY-20250101). All animal experiments adhered to the relevant laws and regulations.

### 2.2. Phenotypic Measurement

We randomly selected 502 male Chengkou Mountain chickens (120 days old) for this study. All the experimental chickens were of the same generation and hatched in the same batch. The experimental chickens were raised at Chengkou Mountain Chicken Genetic Resource Research Institute in Chengkou County (Chongqing, China). The chickens were housed in individual cages under uniform experimental conditions. The diets of the experimental chickens were designed to satisfy the nutrient requirements of chickens [20] and the recommendations of the Chinese chicken feeding standards. The feeding environmental conditions of the experimental chickens at different ages are shown in Table 1.

Following slaughter, 502 breast muscle samples were collected for nucleotide, amino acid, fatty acid, and IMF (intramuscular fat) content measurements. The samples were stored at −20 °C, with analyses conducted by Qingdao Standard Testing Co., Ltd (Qingdao, China). The nucleotide content was determined using an Agilent 1260 liquid chromatography (Agilent Technologies, Santa Clara, CA, USA). The amino acid content was measured according to the Light Industry Standard QB/T 4356-2012 (China Light Industry Standard: determination of free amino acids in Huangjiu by high-performance liquid chromatography) [21], using an Agilent 1260 liquid chromatography (Agilent Technologies, Santa Clara, CA, USA). The fatty acid content was measured according to the National Standard of the People’s Republic of China GB 5009.168-2016 (National Food Safety Standard: determination of fatty acids in foods) [22], using an Agilent 7890A gas chromatography (Agilent Technologies, Santa Clara, CA, USA). The IMF content was measured according to the National Standard of the People’s Republic of China GB 5009.6-2016 (National Food Safety Standard: determination of fat in foods) [23].

### 2.3. Blood Collection and DNA Extraction

Before slaughter, 2 mL of blood was collected from the wing vein of each experimental chicken and frozen at −20 °C. DNA extraction and sequencing were entrusted to China Agricultural University (Beijing, China). DNA was extracted using the magnetic bead method, and the DNA concentration using a Qubit fluorometer (Invitrogen, Carlsbad, CA, USA) and integrity (1% agarose gel electrophoresis) were assessed. Samples of acceptable quality were used for library preparation. When preparing the library, the concentration of each sample was 10 ng/μL, and 50 μL of DNA samples was used. Thus, the amount of DNA used for each sample was 50 ng. A 300–500 bp DNA fragment library was then constructed. After passing the quality control criteria, sequencing was performed.

### 2.4. Low-Depth Resequencing and Accuracy Evaluation

Low-depth resequencing was performed on all individuals. The FastQC software (v0.12.1) was used to assess the quality of the raw sequencing data, examining base quality distribution, GC content, and other indicators to ensure that the data met the requirements for subsequent analysis. The Trimmomatic software (v0.36) was used to remove low-quality reads, with the parameters set to remove windows with fewer than four bases or a mean quality score below 15 to enhance data reliability. Using the field-programmable gate array (FPGA) hardware-accelerated GTX-one computing platform, all sequencing data were aligned to the Gallus6 chicken reference genome using the BWA software (v0.7.17). The aligned results were sorted and indexed using Samtools (v1.17). The BaseVar software (v0.0.1.3) was used to identify polymorphic SNP sites and their population frequencies from low-depth data using maximum likelihood and multiple likelihood ratio tests. This algorithm could detect bi-allelic, tri-allelic, and tetra-allelic SNPs. The filter conditions were set as follows: Depth of coverage (DP) > 1.5, interquartile range (IQR) and effective allele frequency (EAF) > 0.01. Based on the minor allele frequency (MAF) > 0.01, genotype score imputation information score (INFO_SCORE) > 0.4, and call rate > 0.95, the final genotype results were obtained using the search tool for interactions of chemicals (STITCH) method.

Twenty individuals were selected for 20× deep sequencing to evaluate the accuracy of low-depth resequencing. The same FastQC (v0.12.1) and BWA (v0.7.17) alignment workflows were used. After alignment, SNP detection was performed using the GATK software (v4.2.2.0), and a GVCF file was generated for each individual. Joint calling was performed for all samples, generating a chromosome-specific VCF file. The preliminary filtering conditions were fisher strand (FS) > 30 and quality by depth (QD) < 2. Further quality control was performed using VCFtools (v0.1.12), and the genotype results were obtained based on a minor allele frequency (MAF) > 0.01.

### 2.5. GWAS and SNP Annotation

GWAS analysis and SNP annotation followed the method described by Gao et al. [24]. A GWAS was performed using the GEMMA software (v 0.98.1) and the linear mixed model (LMM) as follows: y = Wα + xβ + ε, where y is the phenotypic value for all individuals, W is a covariance matrix used to control the population structure (fixed effects: PC1 and PC2), α is a vector of the corresponding coefficients including the intercept, x is the genotype of the SNP or haplotype marker, β is the effect size of the SNP or haplotype marker for the phenotypes, and ε is a vector of random residuals. Wald test statistics were used to assess the significance of associations between SNPs and phenotypes, and *p*-values were adjusted using Bonferroni correction. The potential and significance association threshold for genome-wide analysis was calculated as *p* = 0.05/N or 1/N, where N is the number of independent SNPs. A total of 2,275,029 SNPs were identified in this study and could be used for subsequent analysis. Thus, the potential or significance threshold of associated SNP in this study is 4.80 × 10^−7^ with—log10^(*p*-value)^ = 6.36 or 2.20 × 10^−8^ with—log10^(*p*-value)^ = 7.66, respectively.

The BEDTools software (v 2.30.0) was used to extract genetic information from the 500 kb upstream and downstream regions of each potential SNP in the genome. SNP annotation was performed using the Annovar software (v 2023-08-05), SnpEff (v 5.1), VEP (v 110), Oncotator (v 1.9.1.0). KEGG (Kyoto Encyclopedia of Genes and Genomes) and GO (Gene Ontology) enrichment analyses were conducted using the OmicShare Tools website (https://www.omicshare.com/tools/, accessed on 10 February 2025).

### 2.6. Haplotype Construction and Association Analysis

Haplotypes were constructed for the significant SNPs annotated to clearly functional genes using Haploview.jar (v4.2). The software Ubuntu 20.04.6 LTS, PLINK (v1.9.0), and VCFtools (v0.1.16) were used to extract the genotypic information of SNPs within the haplotype blocks. The software R (v4.4.2) and RStudio (v2024.09.1) were employed to conduct association analyses among genotypes, haplotypes, and relevant phenotypes.

## 3. Results

### 3.1. Description of Phenotypic Data

**Figure 1:**
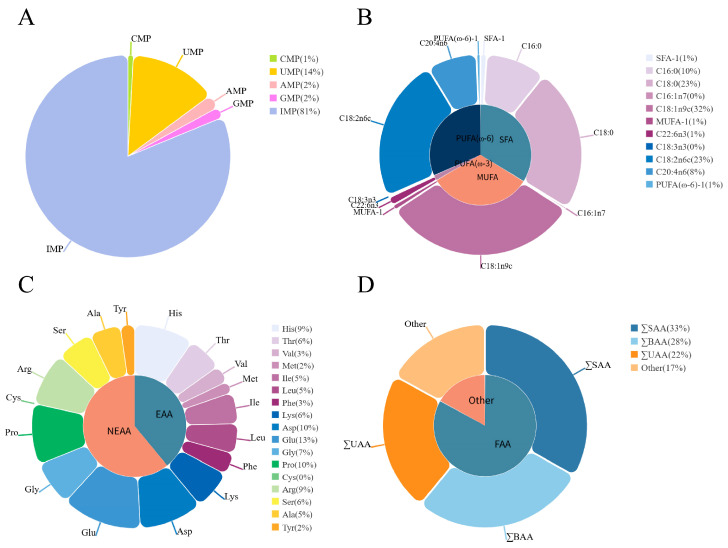
Pie charts of the average content of each flavor precursor substance. (**A**) Pie chart of the average content proportion of nucleotides. CMP: Cytidylic Acid; UMP: Uridylic Acid; AMP: Adenine Nucleotide; GMP: Guanylic Acid; IMP: Inosinic Acid. (**B**) Pie chart of the average content proportion of fatty acids. SFA (saturated fatty acid): C14:0, C16:0, C18:0, C22:0, and C24:0 (Myristic Acid, Palmitic Acid, Stearic Acid, Behenic Acid, and Lignoceric Acid, respectively); MUFA (monounsaturated fatty acid): C16:1n7, C18:1n9c, C20:1, C22:1n9, and C24:1n9 (Palmitoleic Acid, Oleic Acid, Cis-11-Eicosenoic Acid, Erucic Acid, and Nervonic Acid, respectively); PUFA (polyunsaturated fatty acid): C18:3n3, C22:6n3, C18:2n6c, C20:2, C20:3n6, and C20:4n6 (Linolenic Acid, Docosahexaenoic Acid, Linoleic Acid, Eicosadienoic Acid, Dohomo-Γ-Linolenic Acid, and Arachidonic Acid, respectively); PUFA (ω-3) (ω-3 polyunsaturated fatty acid): C18:3n3 and C22:6n3 (Linolenic Acid and Docosahexaenoic Acid, respectively); PUFA (ω-6) (ω-6 polyunsaturated fatty acid): C18:2n6c, C20:2, C20:3n6, and C20:4n6 (Linoleic Acid, Eicosadienoic Acid, Dohomo-Γ-Linolenic Acid, and Arachidonic Acid, respectively). The content of some fatty acids is relatively low and not reflected in the pie chart. Therefore, some fatty acids of the same type were combined. SFA-1 includes C14:0, C22:0, and C24:0; MUFA-1 includes C20:1, C22:1n9, and C24:1n9; PUFA (ω-6)-1 includes C20:2 and C20:3n6. (**C**) Pie chart of the average content proportion of amino acids. EAA (essential amino acid): His, Thr, Val, Met, Ile, Leu, Phe, and Lys (Histidine, Threonine, Valine, Methionine, Isoleucine, Leucine, Phenylalanine, and Lysine, respectively); NEAA (non-essential amino acid): Asp, Glu, Gly, Pro, Cys, Arg, Ser, Ala, and Tyr A (Aspartic Acid, Glutamate, Glycine, Proline, Cystine, Arginine, Serine, Alanine, and Tyrosine, respectively). (**D**) Pie chart of the average content proportion of various flavor amino acids. ∑SAA (sweet amino acid): Thr, Gly, Pro, Ser, and Ala; ∑BAA (bitter amino acid): His, Val, Met, Ile, Leu, and Phe; ∑UAA (umami amino acid): Asp and Glu; FAA (flavor amino acid): sweet, bitter, and umami amino acids.

Five types of umami nucleotides were detected in the breast muscle of the Chengkou mountain chicken, including CMP (Cytidylic Acid), UMP (Uridylic Acid), AMP (Adenine Nucleotide), GMP (Guanylic Acid), and IMP (Inosinic Acid), with IMP having the highest content (Figure 1, Table S1).

The amino acid profile in the breast muscle of the Chengkou mountain chicken is presented in Figure 1 and Table S2. A total of seventeen amino acids were detected, including eight essential and nine non-essential amino acids. Of these, thirteen were flavor amino acids, including five sweet, six bitter, and two umami. The two amino acids present in the highest amounts were glutamic acid and aspartic acid, both of which are umami amino acids.

Figure 1 and Table S3 show the results for intramuscular fat (IMF) and fatty acid content in the breast muscle. The IMF content was 1.5733 g/100 g. Additionally, sixteen free fatty acids were identified, including five saturated fatty acids, five monounsaturated fatty acids, and six polyunsaturated fatty acids. Among the polyunsaturated fatty acids, two were Ꞷ-3 and four were Ꞷ-6 fatty acids. The most abundant fatty acids were oleic acid (C18:1n9c), stearic acid (C18:0), and linoleic acid (C18:2n6).

### 3.2. Correlation Analysis of Traits

**Figure 2:**
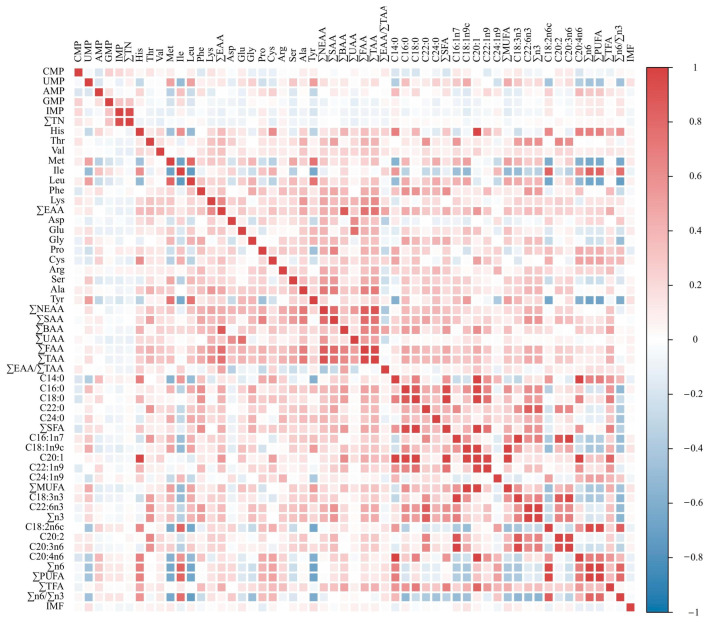
Trait correlation heat map.

Strong correlations were observed between several amino acids and fatty acids. Met, Leu, and Tyr were strongly correlated with Ile. His, Met, Leu, and Tyr were strongly correlated with C18:2n6c, ∑ω-6, and ∑PUFA. Met, Ile, Leu, and Tyr also showed strong correlations with the ratio of ∑ω-6/∑ω-3. C18:0 was strongly positively correlated with C14:0, C16:0, C22:1n9, and C20:4n6. C22:6n3 was strongly or very strongly positively correlated with C16:0, C22:0, C16:1n7, C20:2, and C20:3n6. C22:0, C16:1n7, and C20:2 were strongly positively correlated with C20:3n6. C20:1 exhibited extremely strong positive correlations with several traits, including His, C14:0, C16:0, C18:0, C18:1n9c, C22:1n9, and C20:4n6 (Figure 2, Table S4).

### 3.3. Sequencing Results and Accuracy Evaluation

**Figure 3:**
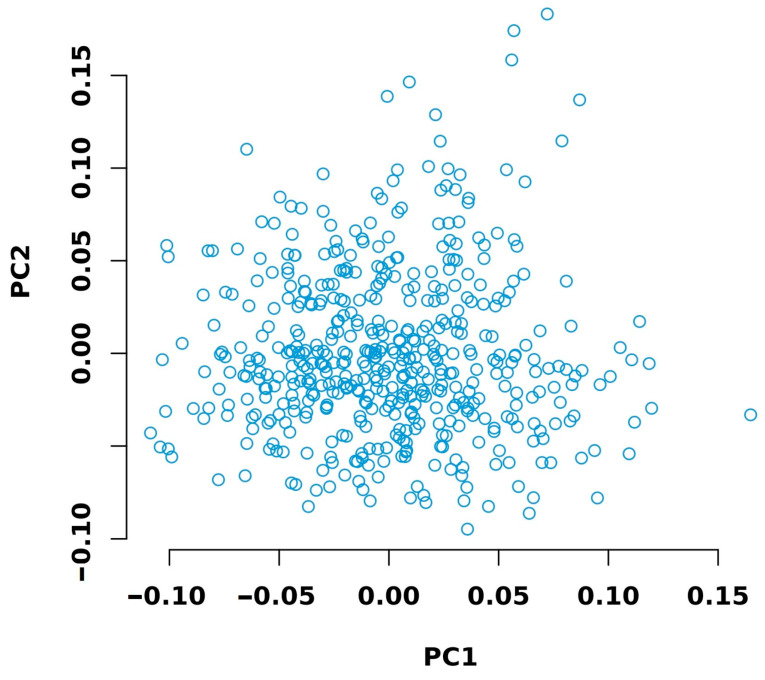
Principal component analysis of the experimental chicken population.

Sequencing was performed for 502 individuals. After data filtering and quality assessment, the average clean data per sample was 0.57 Gb, with an average sequencing depth of 0.5× and an alignment rate of 0.986. The Q20 and Q30 of the sequencing data were 98.56% and 95.76%, respectively. Both values were above 90%, indicating the excellent quality of the sequencing data (Table S5). Principal component analysis revealed no stratification within the chicken population (Figure 3).

To enhance the reliability of the data, this study also selected 20 individuals for sequencing to 20× depth and compared these data with the sequencing data to evaluate the accuracy. Based on 20 samples analyzed using 20× GATK typing and 0.5× STITCH typing, a total of 508,737 SNPs were obtained. When comparing the typing results of chromosome 3 from the two methods, the depth consistency between the two approaches was 0.965 (Table S6).

### 3.4. GWAS and SNP Annotation Results

Principal component analysis (PCA) revealed no stratification within the experimental chicken population (Figure 3). Therefore, it was considered appropriate to proceed with genome-wide association analysis.

**Figure 4:**
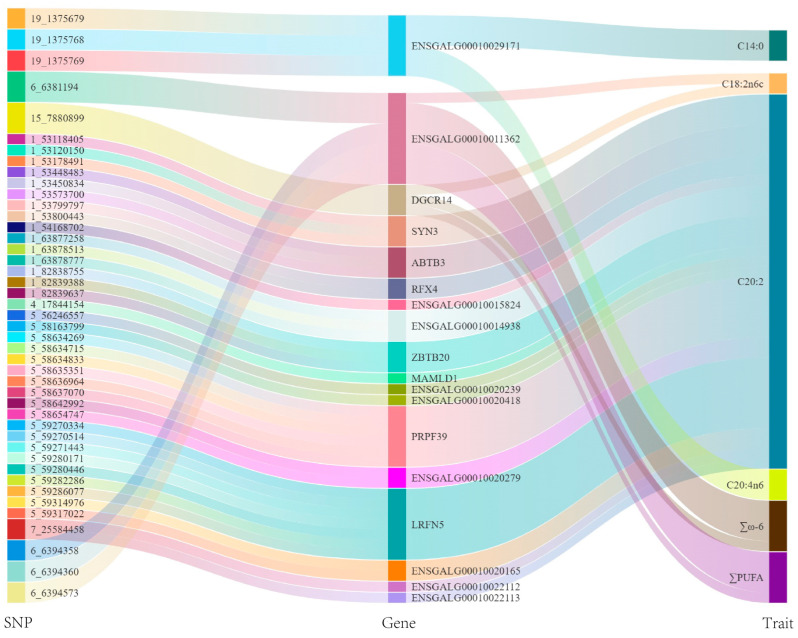
SNP–gene–trait associations revealed in a Sankey plot.

**Figure 5:**
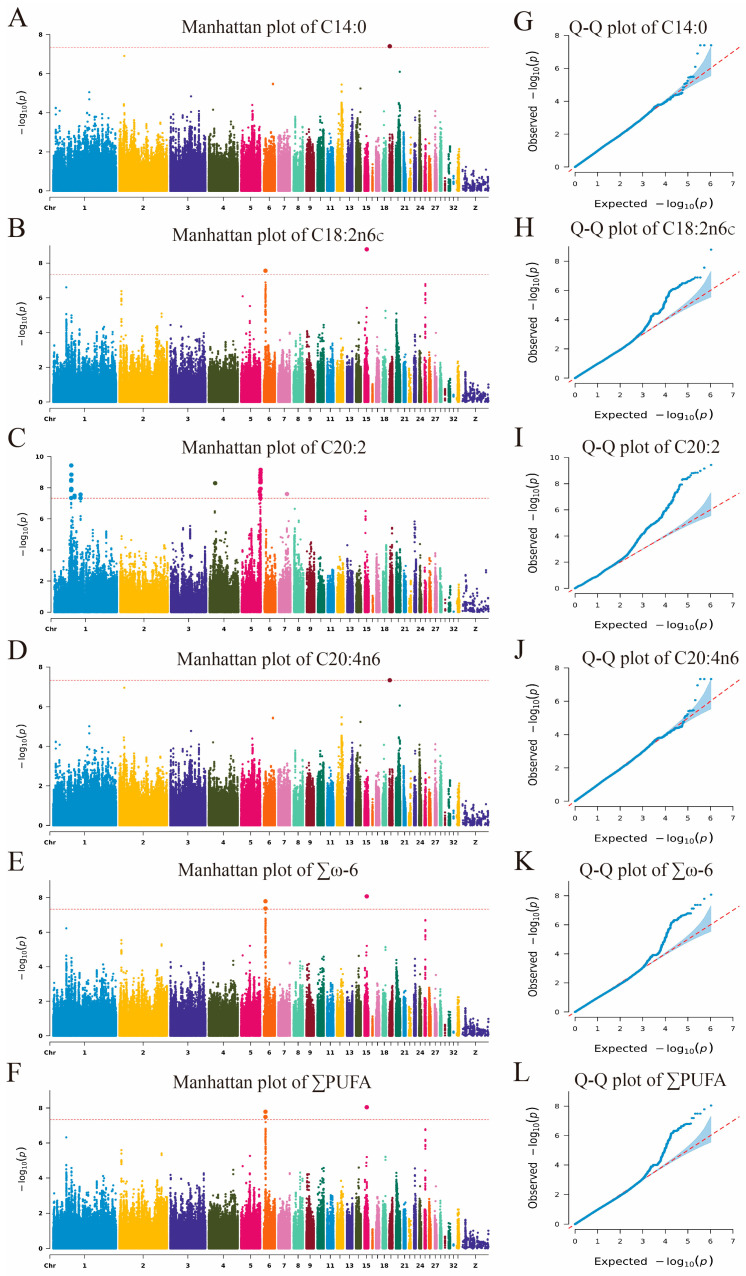
Manhattan and Q-Q plots of GWAS for flavor precursor traits (only traits with potentially or significantly associated SNPs). (**A**–**F**) Manhattan plots; (**G**–**L**) Q-Q plots. The red dotted line means the potential significance threshold.

The GWAS identified 44 SNPs significantly or potentially significantly associated with fatty acid traits, which were mapped to 18 genes across chromosomes 1, 4, 5, 6, 7, 15, and 19. These SNPs were linked to traits such as C14:0, C18:2n6c, C20:2, C20:4n6, ∑ω-6, and ∑PUFA, with 3, 2, 36, 3, 5, and 5 SNPs, respectively (Table S7, Figure 4 and Figure 5). Among them, the five SNPs located on chromosomes 6 and 15 were significantly or potentially significantly associated with the traits ∑ω-6 and ∑PUFA, respectively, and the three SNPs located on chromosome 19 were significantly or potentially significantly associated with the traits C14:0 and C20:4n6.

**Figure 6:**
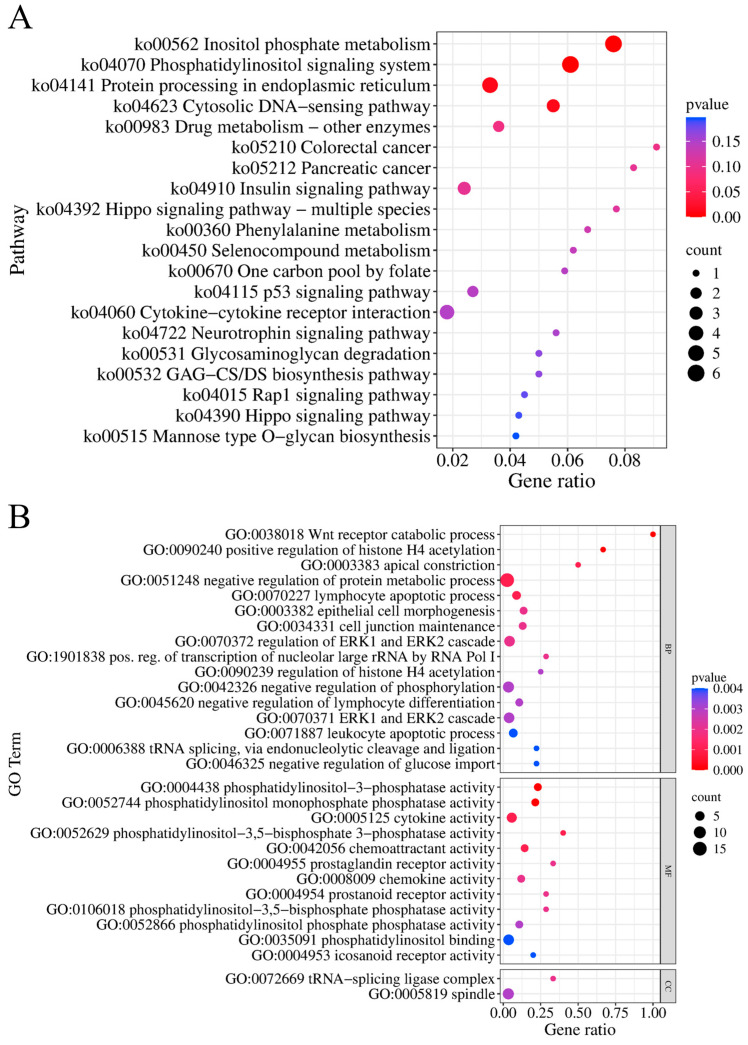
Functional enrichment analysis. (**A**) KEGG enrichment analysis. (**B**) The 30 most significant entries in the GO enrichment analysis (MF: molecular function; BP: biological process; CC: cellular component).

Gene annotation was performed within the 500 kb regions upstream and downstream of each SNP, resulting in 407 genes. Among them, 174 genes were annotated to specific gene names and regarded as candidate genes. Subsequently, functional enrichment analysis was conducted on these 174 genes, and significant enrichment was found in four KEGG pathways and 516 GO terms (*p* < 0.05) (Tables S8 and S9). The four significantly enriched KEGG pathways include inositol phosphate metabolism (ko00562, *p* < 0.001), phosphatidylinositol signaling system (ko04070, *p* < 0.001), protein processing in endoplasmic reticulum (ko04141, *p* = 0.012), and the cytosolic DNA-sensing pathway (ko04623, *p* = 0.013) (Figure 6, Table S8). The top 10 most significant GO terms include Wnt receptor catabolic process (GO:0038018, *p* < 0.001), phosphatidylinositol-3-phosphatase activity (GO:0004438, *p* < 0.001), positive regulation of histone H4 acetylation (GO:0090240, *p* < 0.001), phosphatidylinositol monophosphate phosphatase activity (GO:0052744, *p* < 0.001), apical constriction (GO:0003383, *p* = 0.001), cytokine activity (GO:0005125, *p* = 0.001), phosphatidylinositol-3,5-bisphosphate 3-phosphatase activity (GO:0052629, *p* = 0.001), chemoattractant activity (GO:0042056, *p* = 0.001), negative regulation of protein metabolic process (GO:0051248, *p* = 0.001), and lymphocyte apoptotic processes (GO:0070227, *p* = 0.001) (Figure 6, Table S9).

### 3.5. Association Analysis of Haplotypes with Traits

Haplotype analysis was exclusively conducted on the SNPs that were injected into functional genes. After screening, only the SNPs that had a significant association with C20:2 could undergo haplotype analysis.

**Figure 7:**
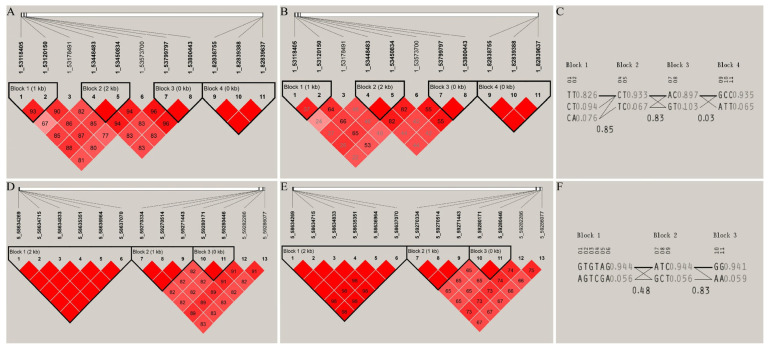
Haplotype construction and linkage disequilibrium analysis of SNPs significantly associated with traits on chromosomes 1 and 5. (**A**–**C**) The D’ value, r^2^ value, and haplotype frequency of the blocks formed by the SNPs on chromosome 1, respectively; (**D**–**F**) the D’ value, r^2^ value, and haplotype frequency of the blocks formed by the SNPs on chromosome 5, respectively.

On chromosome 1, the SNPs significantly associated with C20:2 formed four blocks, which corresponded to the genes *SYN3*, *ABTB3*, *RFX4*, and *ZBTB20*, respectively. Among them, the SNPs corresponding to the genes *ABTB3*, *RFX4*, and *ZBTB20* were completely linked to each other (D’ = 1), and strong linkage disequilibrium was noted between the SNPs 1_53118405 and 1_53120150 corresponding to the *SYN3* gene (D’ > 0.8 and r^2^ > 0.33). On chromosome 5, the SNPs significantly associated with C20:2 formed two blocks, corresponding to the genes *PRPF39* and *LRFN5*, respectively. The SNPs within each block were all completely linked to each other (D’ = 1) (Figure 7).

Statistical analysis was conducted on the SNPs, genotypes, and haplotype distributions within the blocks, and the associations among genotypes, haplotypes, and the C20:2 trait were determined (Table 2 and Table 3). SNP genotype association analysis showed that for SNP 1_53118405, the C20:2 content in the pectoral muscles of individuals with the TT genotype was significantly higher than that of individuals with the CC genotype. For SNP 1_53120150, the C20:2 content of individuals with the TT genotype was significantly higher than that of individuals with the AT and AA genotypes. For SNP 1_53450834, the C20:2 content of individuals with the TT genotype was significantly higher than that of individuals with the CT and CC genotypes (Table 2). Haplotype association analysis showed that the SNPs 1_53448483 and 1_53450834 located in the gene *ABTB3* are within the haplotype block (53,448,483 bp to 53,450,834 bp) on chromosome 1, and they are significantly associated with the C20:2 trait. The C20:2 content of individuals with the CTCT haplotype combination was significantly higher than that of individuals with the TCCCT and TCTC haplotype combinations (Table 3).

## 4. Discussion

### 4.1. Phenotypic Analysis

The flavor nucleotides detected in the breast muscle of Chengkou mountain chicken include CMP, UMP, AMP, GMP, and IMP. Among these, IMP is the most abundant and has a significant content advantage in the breast muscle of Chengkou mountain chicken. The high content of IMP is crucial for the flavor development in Chengkou mountain chicken breast muscle [25]. In this study, the IMP content was 5191.22 mg/kg, which is higher than other breeds such as Yeonsan Ogye [11] and Guangde Rongda poultry [25]. This is potentially attributable to genetic distinctions and environmental adaptations unique to this indigenous breed.

Seventeen amino acids were detected in the breast muscle of Chengkou mountain chicken, which is consistent with the findings of Qie et al. [26]. Among the detected amino acids, glutamic acid and aspartic acid exhibit the highest content, and both of these amino acids are essential components of flavor amino acids. This finding further supports the advantage of Chengkou mountain chicken breast muscle in umami flavor, as glutamic acid and aspartic acid are major contributors to umami flavor [27,28]. In this study, essential amino acids account for 39.29% of the total amino acids, which is lower than that of Tibetan chicken (40.31–40.83%) but close to the WHO/FAO-recommended value for essential amino acids (40.00%) [26]. Additionally, this study found that Thr, Val, Ile, and Leu accounted for 5.48%, 2.74%, 4.92%, and 4.71%, respectively, of total amino acids, whereas the WHO/FAO-recommended values for these essential amino acids are 5.00%, 4.00%, 7.00%, and 4.00% [26], respectively, indicating that some essential amino acids fall short of the recommended levels. These findings highlight that, as a premium poultry breed, Chengkou mountain chicken warrants further investigation into strategies for elevating essential amino acid content in breast muscle tissue, thereby addressing evolving consumer nutritional expectations.

The intramuscular fat (IMF) content in the breast muscle of the Chengkou mountain chicken was found to be 1.5733 g/100 g, which is significantly lower than that in the breast muscle of other chicken breeds [29,30]. Fatty acids are an important component of meat flavor. In this study, 16 types of fatty acids were detected, with oleic acid (C18:1n9c), stearic acid (C18:0), and linoleic acid (C18:2n6) being the most abundant. This is consistent with the findings of Cho et al. [12]. Lee et al. suggested that the ratio of polyunsaturated fatty acids (PUFAs) has minimal impact on chicken meat flavor, with a higher ratio of PUFAs enhancing the umami and savory flavor [31]. The proportion of polyunsaturated fatty acids in the breast muscle of Chengkou mountain chicken to the total fatty acids was 33.54%. This value is lower than that in Korean native chicken (KNC) [12] but higher than in Sovv-500 hybrid broilers [32], further indicating the differences between commercial broilers and local chicken breeds. Additionally, a strong correlation is noted between fatty acids, reflecting the complex interactions during fatty acid metabolism. Furthermore, a strong correlation is noted between fatty acids and amino acids, possibly indicating their joint effect during cooking, which may promote the generation of flavor compounds [31].

### 4.2. GWAS Analysis

In this study, GWAS identified 44 SNPs potentially or significantly associated with fatty acid traits that were localized to 18 genes. Functional analysis revealed several genes related to fatty acid and fat deposition, including *ZBTB20*, *RFX4*, *MAMLD1*, *SYN3*, *ABTB3*, *PRPF39*, *LRFN5*, and *DGCR14*. These eight genes may be candidate genes that affect the fatty acid traits.

In the GWAS analysis, the genotypes of some significant SNPs and haplotypes are also significantly associated with C20:2, including SNPs 1_53118405, 1_53120150, and 1_53450834, as well as the haplotype block composed of SNPs 1_53448483 and 1_53450834. This finding indicates that the results are reliable and robust. It is worth noting that the SNPs that comprise the haplotype block significantly associated with C20:2 are located in the *ABTB3* gene. The ABTB3 protein encoded by the *ABTB3* (ankyrin repeat and BTB domain containing 3) gene belongs to the BTB protein family. Members of this family are typically involved in protein ubiquitination and signal transduction. For example, Cullin3 is an important BTB protein that forms a complex with various E3 ubiquitin ligases to regulate protein degradation [33]. However, whether ABTB3 has similar regulatory functions remains to be further studied. In the research on excessive alcohol intake, it has been reported that *ABTB3* gene expression is significantly upregulated, indicating that *ABTB3* may be related to alcohol metabolism, especially in the state of alcoholism or alcohol dependence [34]. Given that alcohol metabolism mainly occurs in the liver, it can be hypothesized that *ABTB3* may be related to the metabolic function of the liver. However, this has not been proven to date.

In addition to the *ABTB3* gene, the GWAS results for fatty acid traits show that multiple SNPs that are potentially or significantly associated with C20:2 are also located inside or near multiple genes such as *ZBTB20*, *RFX4*, *MAMLD1*, *SYN3*, *PRPF39*, and *LRFN5*. *ZBTB20* (Zinc Finger and BTB Domain Containing 20) is a protein-coding gene that encodes the zinc finger protein that plays important roles in lipid metabolism. In the study by Hsu et al., *ZBTB20* was predicted to be one of the most crucial upstream regulators in the changes in the liver tissue transcriptome of obese mice. Here, the downregulation of *LEPR* and *IGFBP1* mediated by *ZBTB20* is involved in lipid accumulation and inflammation, respectively [35]. *ZBTB20* affects blood lipid levels by regulating the expression of the lipoprotein lipase (*LPL*) gene. The study by Li et al. showed that in mice with liver-specific knockout of *ZBTB20*, LPL mRNA and protein levels increased significantly, thus enhancing the plasma triglyceride clearance rate and reducing plasma triglyceride levels [36]. Liu et al. found that in a mouse model of non-alcoholic fatty liver disease (NAFLD) induced by a high-fat diet, significant upregulation of *ZBTB20* expression led to an increase in the content of intrahepatic triglycerides, whereas *ZBTB20* deletion significantly improved the hepatic steatosis and insulin resistance induced by a high-fat diet [37]. These research results indicate that *ZBTB20* maintains the balance of lipid metabolism by regulating the processes of fatty acid synthesis and decomposition. The *LRFN5* (leucine-rich repeat and fibronectin type III domain-containing protein 5) gene encodes a protein related to neuronal growth and synaptic function [38]. Fatty acids play an important role in the nervous system. Wani et al. showed that the deficiency of ω-3 fatty acids may be associated with an increase in depressive symptoms [39]. The changes in *LRFN5* gene expression in various diseases have also been studied. For example, *LRFN5* gene expression is significantly downregulated in NAFLD [40]. It is hypothesized that *LRFN5* is related to lipid metabolism, but functional verification is still needed.

An SNP on chromosome 15 is significantly associated with C18:2n6c, ∑ω-6, and ∑PUFA. This SNP is located near the gene *DGCR14*. The *DGCR14* (DiGeorge syndrome critical region gene 14) gene is associated with a variety of developmental disorders and diseases [41]. Studies have shown that *DGCR14* is highly expressed in the cerebral cortex, especially in the basal ganglia region, and its deletion or dysfunction may lead to abnormal neuronal function. Moreover, it may play a crucial role in neurodevelopment and mental diseases [42]. However, there is currently no research on the connection between *DGCR14* and fatty acids. Further functional verification is needed to determine whether *DGCR14* has an impact on fatty acids.

### 4.3. Gene Function Enrichment Analysis

Kyoto Encyclopedia of Genes and Genomes (KEGG) and Gene Ontology (GO) functional enrichment analysis of the candidate genes for fatty acid traits showed multiple enrichments in phosphatidylinositol-related pathways and terms. Phosphatidylinositol is a molecule composed of inositol and diacylglycerol. Phosphatidylinositol and its derivatives possess multiple functions in the regulation of fatty acid lipids, including regulating lipid metabolism, signal transduction, membrane structure, and cellular functions. During the process of fatty acid metabolism, saturated fatty acids (such as palmitic acid) can influence cellular functions by regulating the synthesis and degradation of phosphatidylinositol. For instance, palmitic acid affects mitochondrial function and autophagy by activating phosphatidylinositol-related pathways [43]. Phosphatidylinositol can also form various derivatives through phosphorylation, such as phosphatidylinositol 4-phosphate (PI4P), phosphatidylinositol 3-phosphate (PI3P), and phosphatidylinositol 4,5-bisphosphate (PI4, 5P_2_). These derivatives play important roles in cell signaling, membrane remodeling, and lipid metabolism. Phosphatidylinositol 4,5-bisphosphate (PI4, 5P_2_) is a key intermediate in the synthesis of glycerophospholipids and is involved in glycerophospholipid storage and transport [44]. Phosphatidylinositol 3,4,5-trisphosphate (PIP_3_) is a critical molecule in insulin signaling. By activating downstream effectors such as protein kinase B (Akt), it regulates cellular metabolism and lipid synthesis. This process is particularly important in obesity and metabolic syndrome because abnormal PIP_3_ levels may lead to insulin resistance [45]. Phosphatidylinositol and its derivatives can indirectly affect the metabolism and distribution of fatty acids by regulating membrane fluidity, ion channel activity, and cytoskeleton reorganization. For example, the localization of phosphatidylinositol 4-phosphate (PI4P) on the cell membrane determines the distribution and function of membrane proteins [46]. The dysregulation of phosphatidylinositol and its derivatives is also associated with various diseases. In diabetes, phosphatidylinositol 3-kinase (PI3K) activation may lead to an increase in fatty acid synthesis [47].

### 4.4. Potential Application of the Research Results

The 44 SNPs identified in this study were significantly associated with fatty acid traits. In addition, the eight candidate genes have important potential application value in marker-assisted selection (MAS) and genomic prediction models.

In terms of marker-assisted selection, these significantly associated SNPs can be used as molecular markers to screen Chengkou mountain chickens with excellent fatty acid traits. For example, for those breeding goals that aim to increase the content of polyunsaturated fatty acids in chicken to improve flavor, the early selection of chicken flocks can be performed based on SNP genotypes related to polyunsaturated fatty acids to accelerate the breeding process and enhance breeding efficiency. Using MAS, a variety of Chengkou mountain chicken with a fatty acid composition that better meets consumers’ demands can be cultivated in a relatively short period of time, enhancing its market competitiveness.

In the construction of genomic prediction models, these SNPs and genetic information can serve as important predictors. Based on the GWAS results, combined with other genetic markers and environmental factors, a more accurate genomic prediction model was constructed to precisely predict the fatty acid traits of Chengkou mountain chickens. This is helpful for evaluating the fatty acid traits of individuals in the early stage of breeding, planning breeding programs in advance, optimizing breeding decisions, avoiding blind breeding, and thereby reducing breeding costs.

### 4.5. Research Limitations

Although this research achieved certain results, the study’s limitations should be noted. First, the GWAS performed in this study only detected SNPs with significant associations with some fatty acid traits of Chengkou mountain chickens and did not cover all the flavor precursors that might affect chicken flavor. Second, although multiple candidate genes have been identified, research on the specific molecular mechanisms of these genes in regulating fatty acid traits is lacking. For example, although the ABTB3 gene is related to C20:2, its detailed regulatory pathway and mode of action in fatty acid metabolism have not yet been clarified. At present, there is also a lack of direct functional verification regarding the relationship between the *DGCR14* gene and fatty acids. Furthermore, this study mainly focuses on the genetic level, and there are relatively few studies on how environmental factors and gene interactions jointly affect the fatty acid traits of Chengkou mountain chickens. In actual production, environmental factors (such as feeding methods, feed composition, etc.) have significant influences on fatty acid composition in chickens. However, this study did not fully consider the complex interactions among these factors and genes, which may lead to an incomplete analysis of the genetic mechanism of fatty acid traits. Future research needs to further expand the scope of study and deeply explore gene functions and environment–gene interactions to improve the understanding and regulatory mechanism of flavor precursor substances in Chengkou mountain chickens.

## 5. Conclusions

In this study, a GWAS allowed for the identification of 44 SNPs potentially or significantly associated with fatty acid traits, localized to chromosomes 1, 4, 5, 6, 7, 15, and 19. These SNPs were further verified using haplotype analysis. According to functional analysis, eight candidate genes were selected, among which *ZBTB20*, *RFX4*, *MAMLD1*, *SYN3*, *ABTB3*, *PRPF39*, *LRFN5*, and *DGCR14* potentially represent key genes affecting fatty acids. This study revealed the functions of the regulated genes, thus playing a key role in elucidating the genes related to flavor precursors in chicken breast muscle.

## Figures and Tables

**Table 1:** The environmental breeding conditions of experimental chickens at different stages.

Age in Days	Temperature	Humidity	Lighting
Incubation period	37–38 °C	65%	-
0–7 d	35 °C	60%	24 h
8–20 d	30 °C	60%	24 h
21–30 d	26 °C	60%	18 h
31–60 d	26 °C	56%	16 h
61–90 d	20 °C	56%	16 h
91–120 d	20 °C	56%	16 h

**Table 2:** Association analysis of SNP genotypes and traits for constructing haplotype blocks.

Trait	SNP	Phenotype (Genotype)			*p*-Value
C20:2	1_53118405	0.0097 ± 0.0033 ^a^ (TT)	0.0092 ± 0.0029 ^ab^ (CT)	0.0079 ± 0.0024 ^b^ (CC)	0.026 *
	1_53120150	0.0096 ± 0.0032 ^a^ (TT)	0.0089 ± 0.0032 ^b^ (AT)	0.0073 ± 0.0020 ^b^ (AA)	0.032 *
	1_53448483	0.0096 ± 0.0033 ^a^ (CC)	0.0089 ± 0.0026 ^a^ (TC)	0.0069 ± 0.0022 ^a^ (TT)	0.068
	1_53450834	0.0096 ± 0.0033 ^a^ (TT)	0.0087 ± 0.0027 ^b^ (CT)	0.0069 ± 0.0022 ^b^ (CC)	0.025 *
	1_53799797	0.0096 ± 0.0033 ^a^ (AA)	0.0094 ± 0.0027 ^a^ (GA)	0.0077 ± 0.0042 ^a^ (GG)	0.689
	1_53800443	0.0095 ± 0.0033 ^a^ (CC)	0.0094 ± 0.0027 ^a^ (TC)	0.0077 ± 0.0042 ^a^ (TT)	0.695
	1_82838755	0.0097 ± 0.0032 ^a^ (GG)	0.0089 ± 0.0028 ^a^ (AG)	/	0.135
	1_82839388	0.0096 ± 0.0032 ^a^ (CC)	0.0089 ± 0.0028 ^a^ (TC)	/	0.147
	1_82839637	0.0096 ± 0.0032 ^a^ (CC)	0.0090 ± 0.0028 ^a^ (TC)	/	0.192
	5_58634269	0.0096 ± 0.0032 ^a^ (GG)	0.0090 ± 0.0029 ^a^ (AG)	/	0.359
	5_58634715	0.0096 ± 0.0032 ^a^ (TT)	0.0090 ± 0.0029 ^a^ (GT)	/	0.359
	5_58634833	0.0096 ± 0.0032 ^a^ (GG)	0.0090 ± 0.0029 ^a^ (TG)	/	0.359
	5_58635351	0.0096 ± 0.0032 ^a^ (TT)	0.0089 ± 0.0029 ^a^ (CT)	/	0.312
	5_58636964	0.0096 ± 0.0032 ^a^ (AA)	0.0089 ± 0.0029 ^a^ (GA)	/	0.306
	5_58637070	0.0096 ± 0.0032 ^a^ (GG)	0.0089 ± 0.0029 ^a^ (AG)	/	0.295
	5_59270334	0.0096 ± 0.0032 ^a^ (AA)	0.0088 ± 0.0030 ^a^ (GA)	/	0.166
	5_59270514	0.0096 ± 0.0032 ^a^ (TT)	0.0088 ± 0.0030 ^a^ (CT)	/	0.172
	5_59271443	0.0096 ± 0.0032 ^a^ (CC)	0.0088 ± 0.0030 ^a^ (TC)	/	0.172
	5_59280171	0.0096 ± 0.0032 ^a^ (GG)	0.0089 ± 0.0031 ^a^ (AG)	/	0.107
	5_59280446	0.0096 ± 0.0032 ^a^ (GG)	0.0089 ± 0.0031 ^a^ (AG)	/	0.107

Note: Different letters in the same row indicate a significant difference between the genotypes (*p* < 0.05 and the upper right corner of the *p* value is marked with *); the same letter indicates no significant difference between them.

**Table 3:** Association analysis of haplotype combinations and traits.

Trait	Gene	Haplotype Combination	Number	Phenotype	*p*-Value
C20:2	*SYN3*	LD1 (TTTT)	284	0.0098 ± 0.0033 ^a^	0.072
		LD2 (CTTT)	67	0.0093 ± 0.0028 ^a^	
		LD3 (CATT)	51	0.0091 ± 0.0032 ^a^	
		LD4 (CACT)	9	0.0080 ± 0.0027 ^a^	
		LD5 (CACA)	4	0.0073 ± 0.0020 ^a^	
	*ABTB3*	LD1 (CTCT)	370	0.0096 ± 0.0032 ^a^	0.024 *
		LD2 (TCCT)	48	0.0087 ± 0.0027 ^b^	
		LD3 (TCTC)	3	0.0069 ± 0.0022 ^b^	
	*RFX4*	LD1 (ACAC)	333	0.0096 ± 0.0033 ^a^	0.689
		LD2 (GTAC)	81	0.0094 ± 0.0027 ^a^	
		LD3 (GTGT)	4	0.0077 ± 0.0042 ^a^	
	*ZBTB20*	LD1 (GCCGCC)	374	0.0097 ± 0.0032 ^a^	0.178
		LD2 (ATTGCC)	54	0.0090 ± 0.0028 ^a^	
	*PRPF39*	LD1 (GTGTAGGTGTAG)	376	0.0096 ± 0.0032 ^a^	0.310
		LD2 (AGTCGAGTGTAG)	45	0.0089 ± 0.0029 ^a^	
	*LRFN5*	LD1 (ATCATC)	387	0.0096 ± 0.0032 ^a^	0.166
		LD2 (GCTATC)	47	0.0088 ± 0.0030 ^a^	
		LD1 (GGGG)	384	0.0096 ± 0.0032 ^a^	0.107
		LD2 (AAGG)	51	0.0089 ± 0.0030 ^a^	

Note: Different letters in the same row indicate a significant difference between the genotypes (*p* < 0.05 and the upper right corner of the *p* value is marked with *); the same letter indicates no significant difference between them.

## Data Availability

All relevant data of this study are presented in the manuscript and its Supplementary Files.

## Supplementary Materials

The following supporting information can be downloaded at: https://www.mdpi.com/article/10.3390/ani15121726/s1. Figure S1: Histogram of the frequency distribution of nucleotide traits; Figure S2: Histogram of the frequency distribution of amino acid traits; Figure S3: Histogram of the frequency distribution of fatty acids and IMF traits; Figure S4: Manhattan and Q-Q plots of GWAS for nucleotides; Figure S5: Manhattan and Q-Q plots of GWAS for amino acids (Part 1); Figure S6: Manhattan and Q-Q plots of GWAS for amino acids (Part 2); Figure S7: Manhattan and Q-Q plots of GWAS for amino acids (Part 3); Figure S8: Manhattan and Q-Q plots of GWAS for amino acids (Part 4); Figure S9: Manhattan and Q-Q plots of GWAS for fatty acids and IMF (Part 1); Figure S10: Manhattan and Q-Q plots of GWAS for fatty acids and IMF (Part 2); Figure S11: Manhattan and Q-Q plots of GWAS for fatty acids and IMF (Part 3); Table S1: Descriptive statistical results of nucleotide content in breast muscle of 120-day-old Chengkou mountain chickens (unit: mg/kg); Table S2: Descriptive statistical results of amino acid content in breast muscle of 120-day-old Chengkou mountain chickens (unit: g/100 g); Table S3: Descriptive statistical results of fatty acid and intramuscular fat content in breast muscle of 120-day-old Chengkou mountain chickens (unit: g/100 g); Table S4: Trait correlation matrix; Table S5: WGS data of 502 individuals; Table S6: Comparison and genotyping consistency of SNP on chromosome 3 between low-depth sequencing and 20× depth sequencing; Table S7: SNPs potentially or significantly associated with traits; Table S8: KEGG significantly enriched pathways; Table S9: GO significantly enriched entries.
