# Supplementary material for: Genome-Wide Association Analysis of Flavor Precursor Traits in Chengkou Mountain Chicken
Source: Animals (Basel). 2025 Jun 11;15(12):1726. doi: 10.3390/ani15121726 (PMC12189354; doi:10.3390/ani15121726)
Supplement: Supplementary file 1 [file animals-15-01726-s001.zip › Supplementary Figure.pdf]

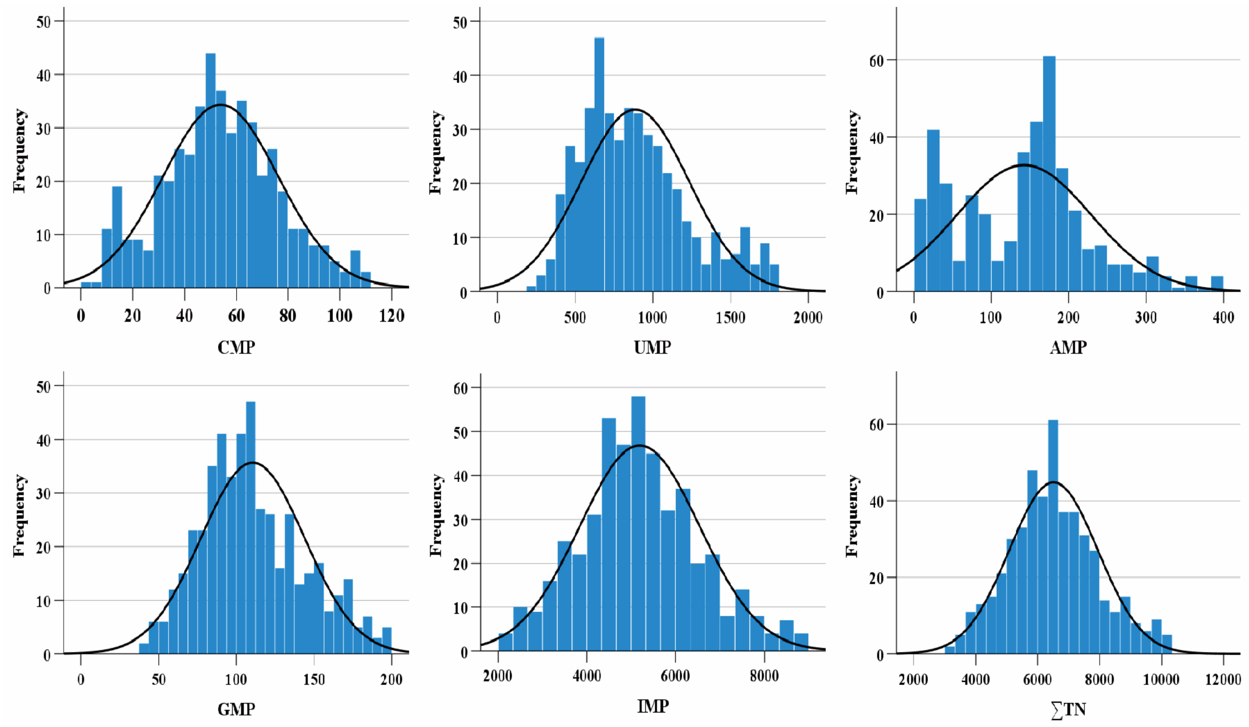

Figure S1. Histogram of the frequency distribution of nucleotide traits.

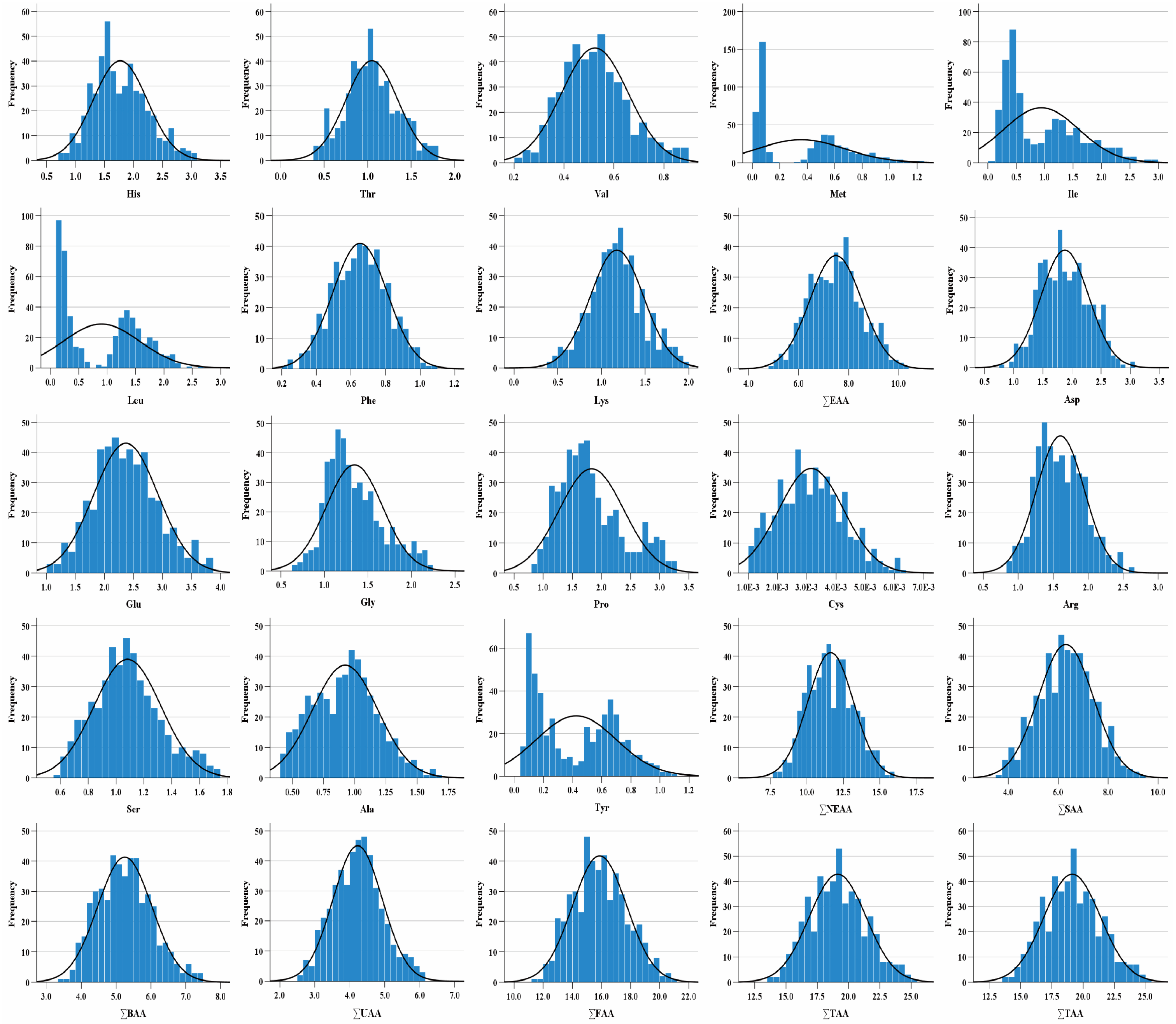

Figure S2. Histogram of the frequency distribution of amino acid traits.

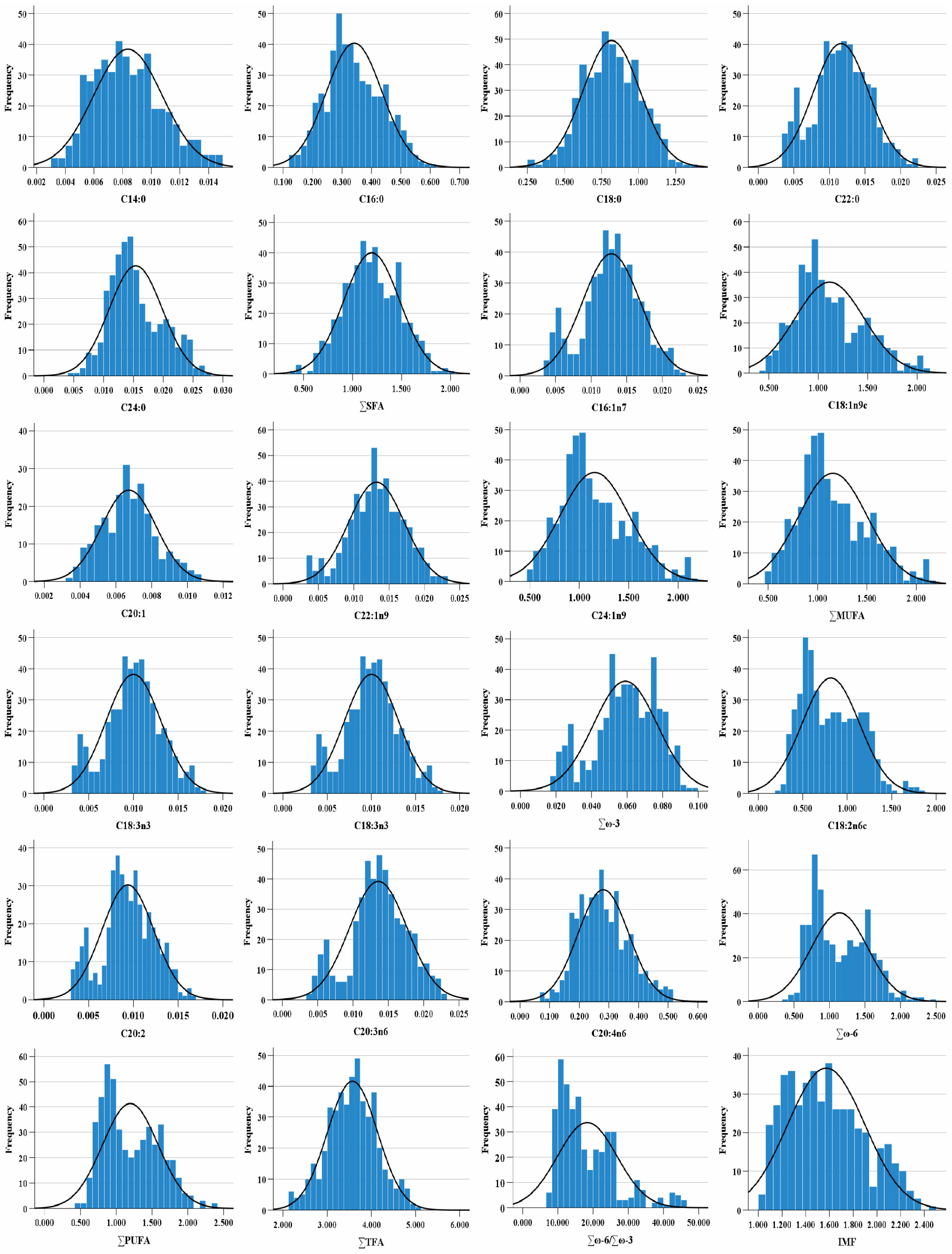

Figure S3. Histogram of the frequency distribution of fatty acids and IMF traits.

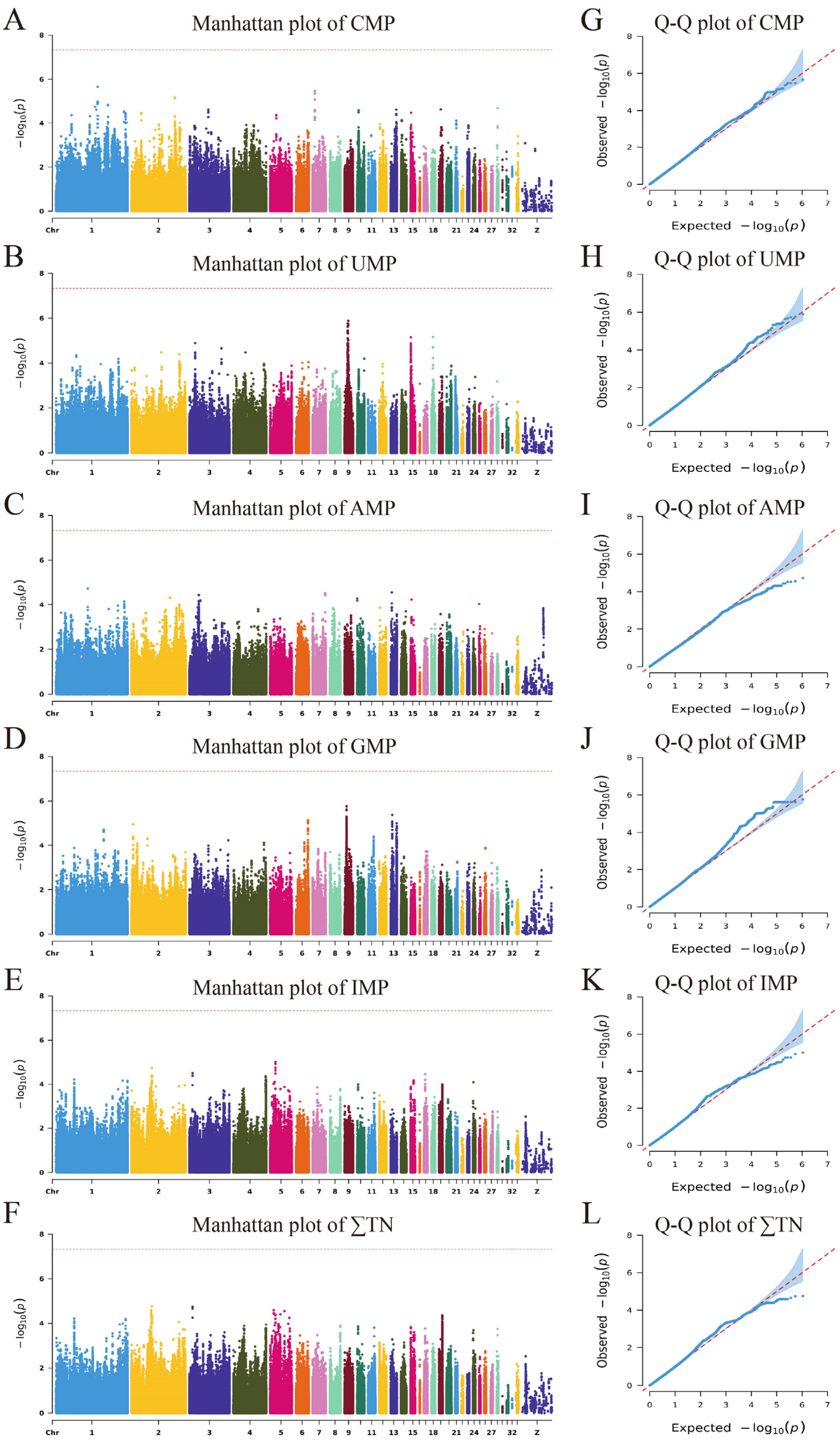

Figure S4. Manhattan and Q-Q plots of GWAS for nucleotides.

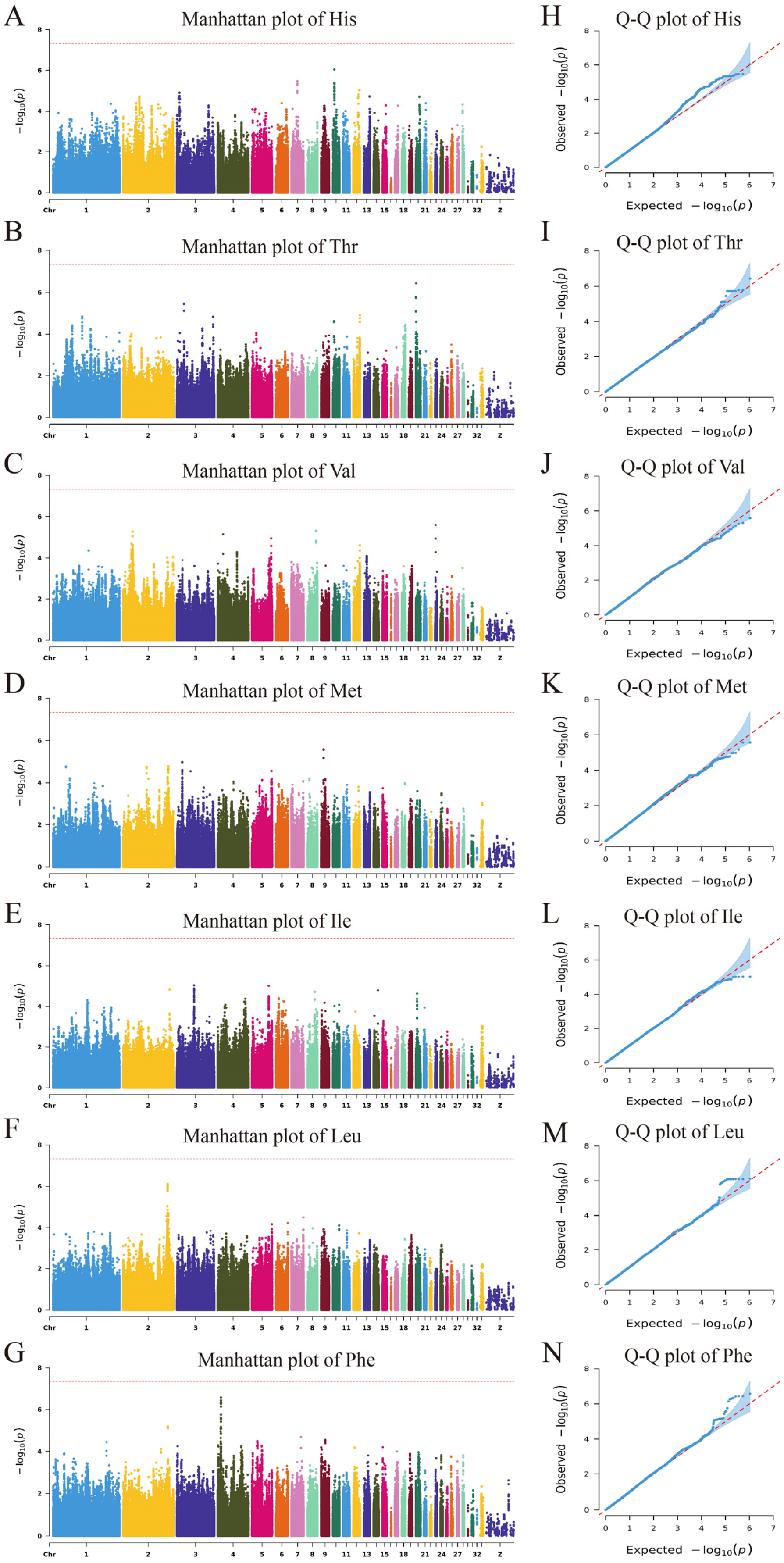

Figure S5. Manhattan and Q-Q plots of GWAS for amino acids (Part 1).

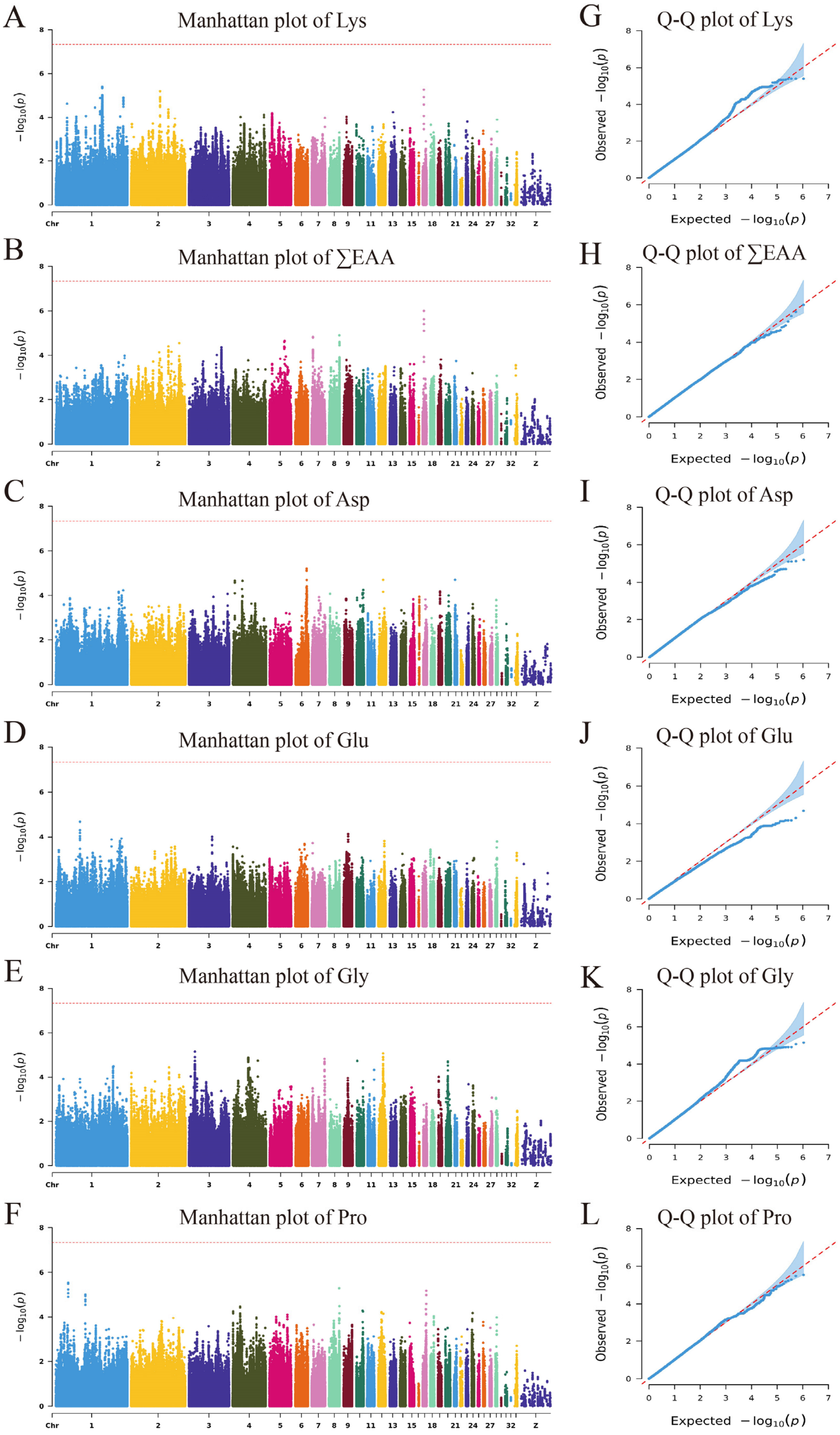

Figure S6. Manhattan and Q-Q plots of GWAS for amino acids (Part 2).

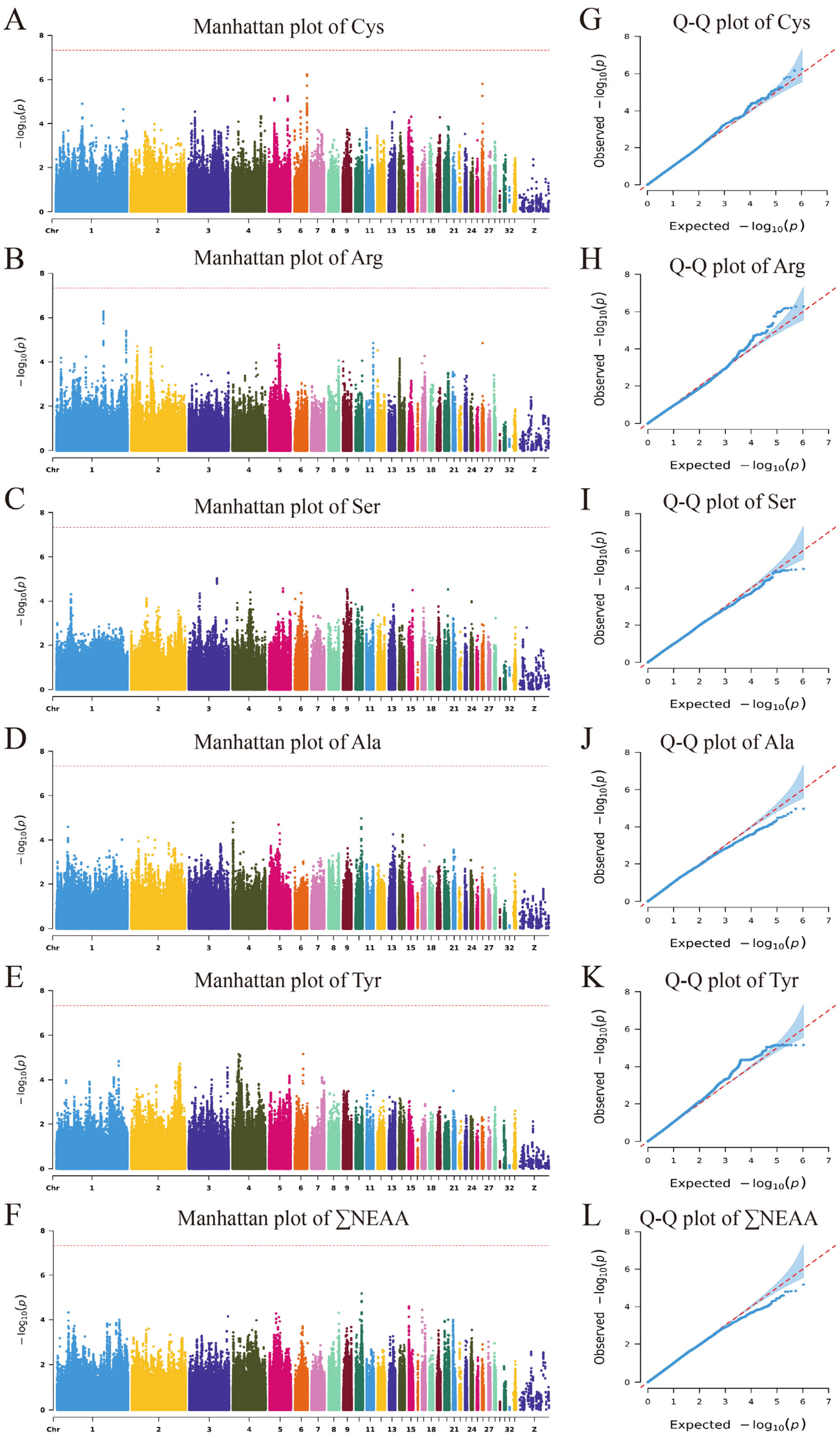

Figure S7. Manhattan and Q-Q plots of GWAS for amino acids (Part 3).

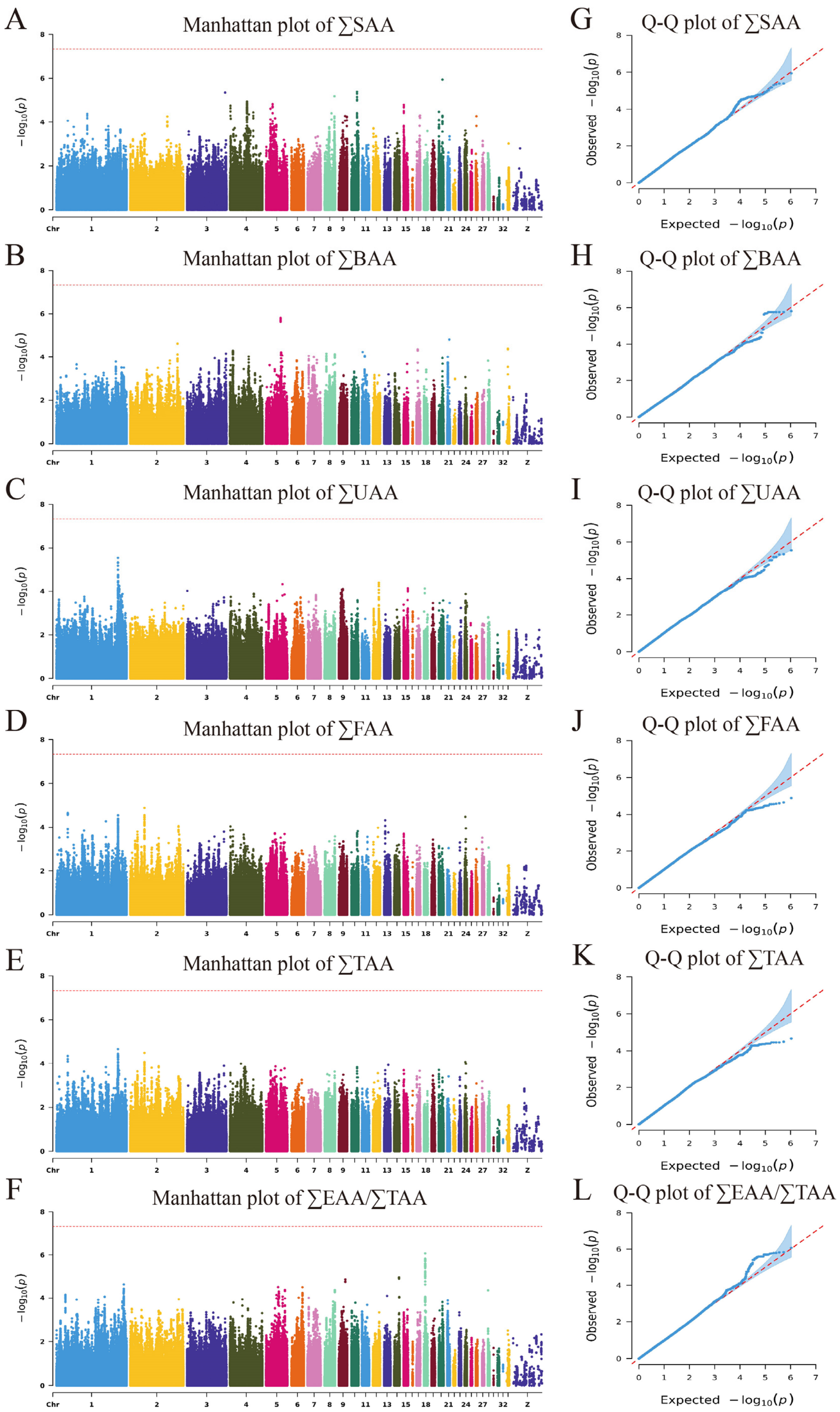

Figure S8. Manhattan and Q-Q plots of GWAS for amino acids (Part 4).

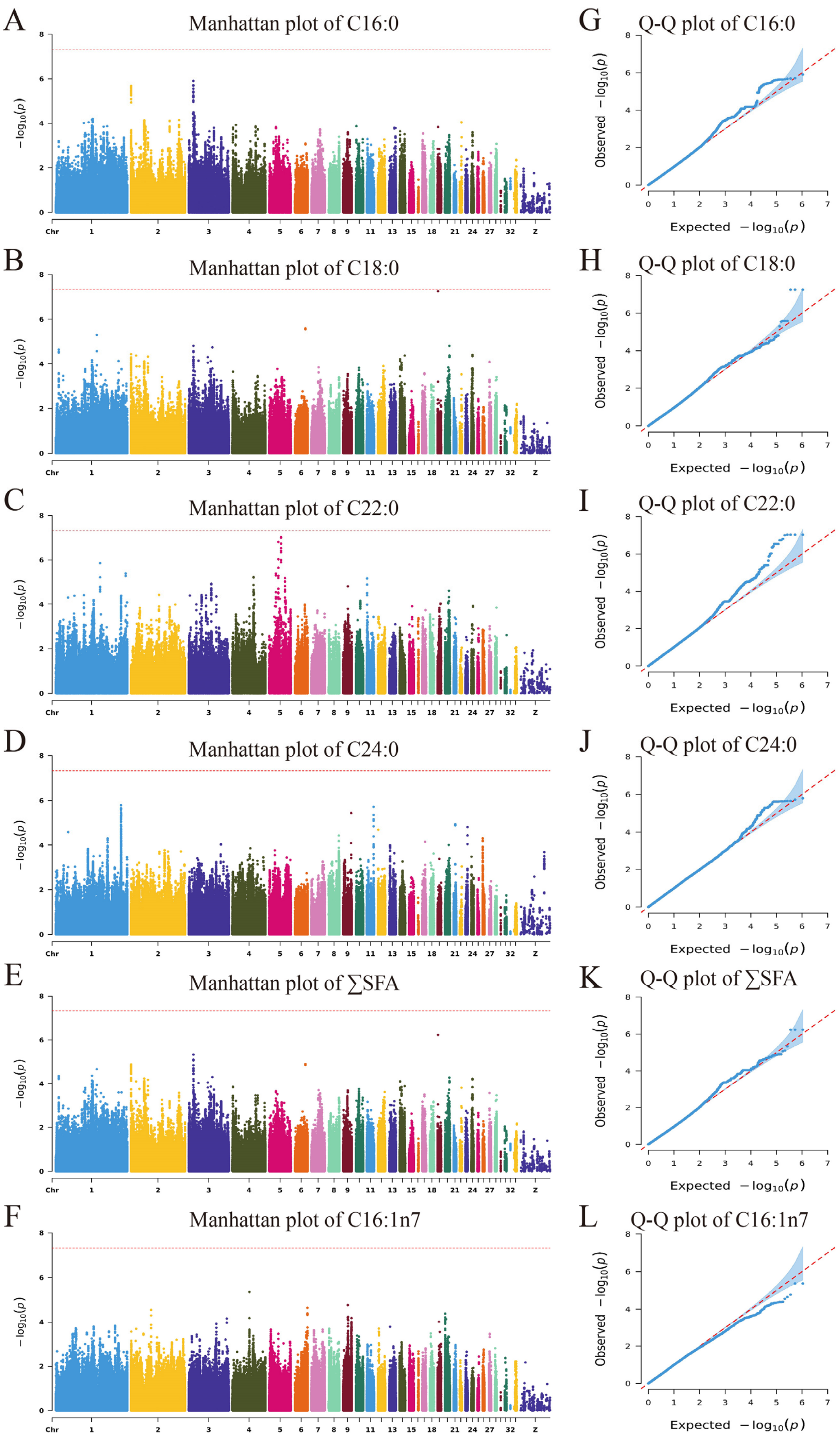

Figure S9. Manhattan and Q-Q plots of GWAS for fatty acids and IMF (Part 1).

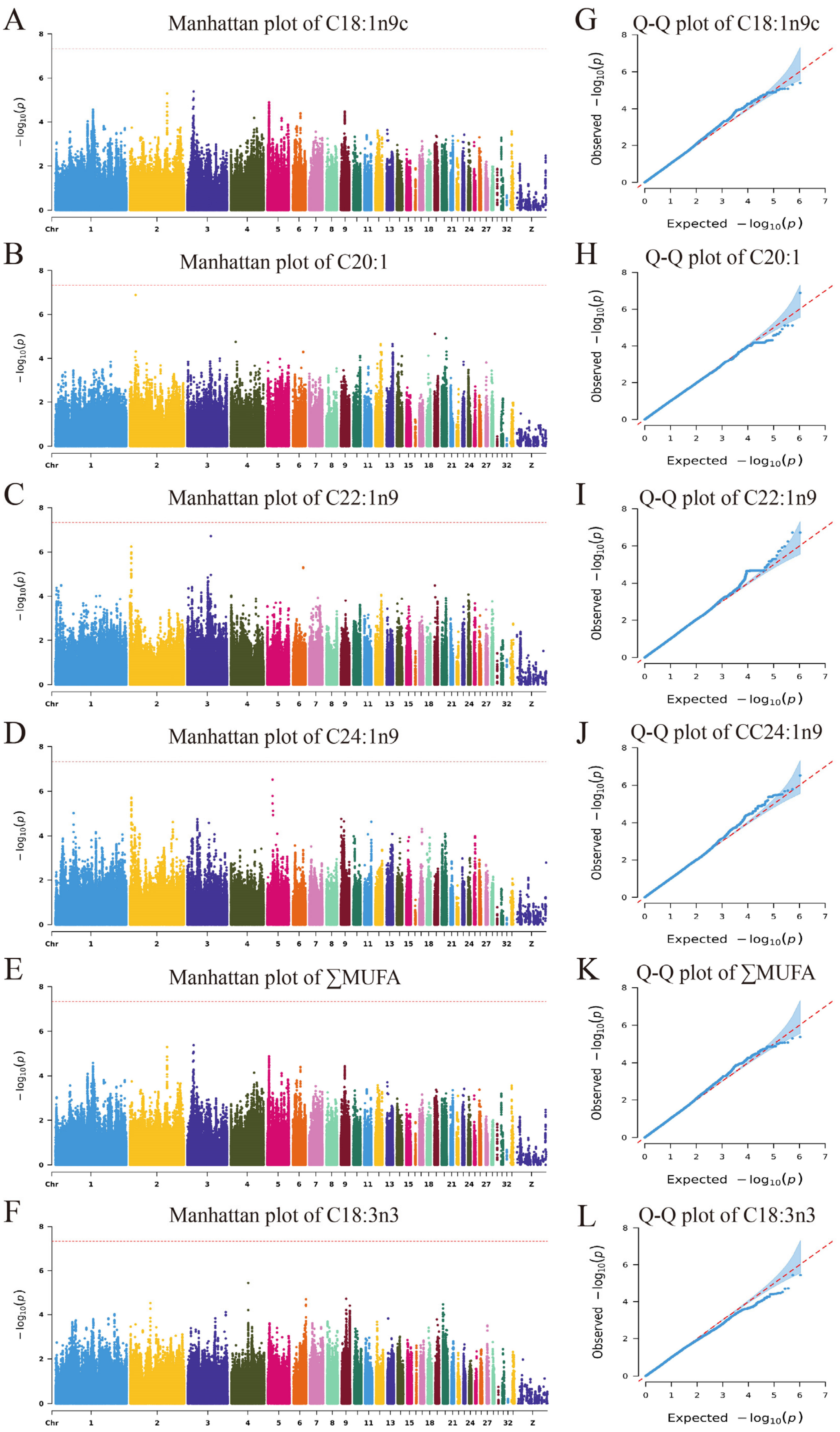

Figure S10. Manhattan and Q-Q plots of GWAS for fatty acids and IMF (Part 2).

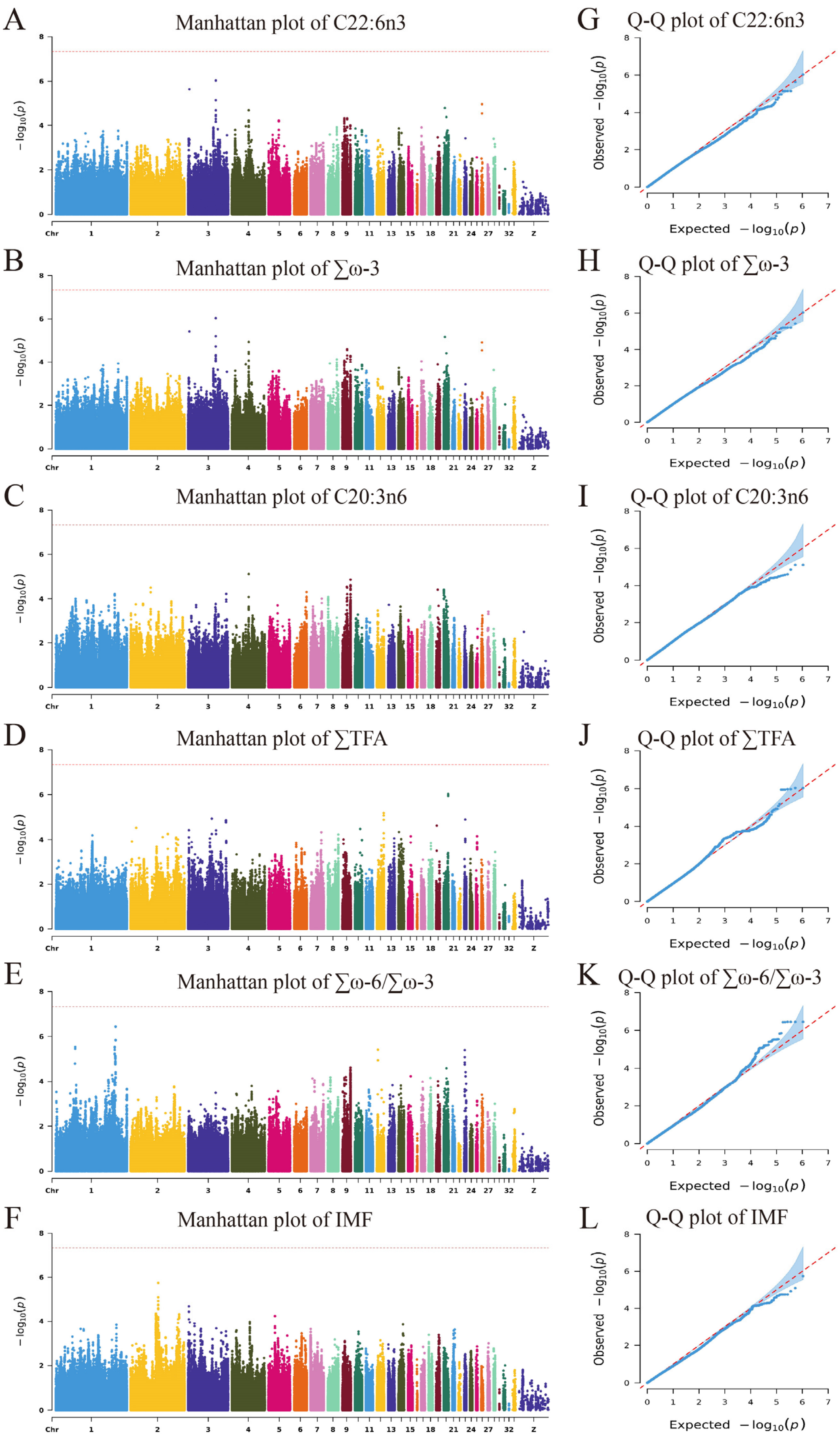

Figure S11. Manhattan and Q-Q plots of GWAS for fatty acids and IMF (Part 3).
